# Supplementary material for: Stenotrophomonas maltophilia Infections: A Systematic Review and Meta-Analysis of Comparative Efficacy of Available Treatments, with Critical Assessment of Novel Therapeutic Options
Source: Antibiotics (Basel). 2023 May 15;12(5):910. doi: 10.3390/antibiotics12050910 (PMC10215754; doi:10.3390/antibiotics12050910)
Supplement: Supplementary file 1 [file antibiotics-12-00910-s001.zip › antibiotics-2372410-supplementary.pdf]

antibiotics

MDPI

Table S1. PRISMA 2020 (Preferred Reporting Items for Systematic Review and Meta-Analysis) checklist.

Section and Topic	Item #	Checklist item	Reported on page #
**TITLE**			
Title	1	Identify the report as a systematic review.	1
**ABSTRACT**			
Abstract	2	See the PRISMA 2020 for Abstracts checklist.	1-2
**INTRODUCTION**			
Rationale	3	Describe the rationale for the review in the context of existing knowledge.	3
Objectives	4	Provide an explicit statement of the objective(s) or question(s) the review addresses.	3
**METHODS**			
Eligibility criteria	5	Specify the inclusion and exclusion criteria for the review and how studies were grouped for the syntheses.	4
Information sources	6	Specify all databases, registers, websites, organisations, reference lists and other sources searched or consulted to identify studies. Specify the date when each source was last searched or consulted.	4, Figure 1
Search strategy	7	Present the full search strategies for all databases, registers and websites, including any filters and limits used.	Table S2
Selection process	8	Specify the methods used to decide whether a study met the inclusion criteria of the review, including how many reviewers screened each record and each report retrieved, whether they worked independently, and if applicable, details of automation tools used in the process.	4
Data collection process	9	Specify the methods used to collect data from reports, including how many reviewers collected data from each report, whether they worked independently, any processes for obtaining or confirming data from study investigators, and if applicable, details of automation tools used in the process.	4
Data items	10a	List and define all outcomes for which data were sought. Specify whether all results that were compatible with each outcome domain in each study were sought (e.g. for all measures, time points, analyses), and if not, the methods used to decide which results to collect.	4
	10b	List and define all other variables for which data were sought (e.g. participant and intervention characteristics, funding sources). Describe any assumptions made about any missing or unclear information.	4
Study risk of bias assessment	11	Specify the methods used to assess risk of bias in the included studies, including details of the tool(s) used, how many reviewers assessed each study and whether they worked independently, and if applicable, details of automation tools used in the process.	5
Effect measures	12	Specify for each outcome the effect measure(s) (e.g. risk ratio, mean difference) used in the synthesis or presentation of results.	5
Synthesis methods	13a	Describe the processes used to decide which studies were eligible for each synthesis (e.g. tabulating the study intervention characteristics and comparing against the planned groups for each synthesis (item #5)).	5-6
	13b	Describe any methods required to prepare the data for presentation or synthesis, such as handling of missing summary statistics, or data conversions.	5-6
	13c	Describe any methods used to tabulate or visually display results of individual studies and syntheses.	5-6
	13d	Describe any methods used to synthesize results and provide a rationale for the choice(s). If meta-analysis was performed, describe the model(s), method(s) to identify the presence and extent of statistical heterogeneity, and software package(s) used.	5-6
	13e	Describe any methods used to explore possible causes of heterogeneity among study results (e.g. subgroup analysis, meta-regression).	5
	13f	Describe any sensitivity analyses conducted to assess robustness of the synthesized results.	5
Reporting bias assessment	14	Describe any methods used to assess risk of bias due to missing results in a synthesis (arising from reporting biases).	5
Certainty assessment	15	Describe any methods used to assess certainty (or confidence) in the body of evidence for an outcome.	Not available
**RESULTS**			
Study selection	16a	Describe the results of the search and selection process, from the number of records identified in the search to the number of studies included in the review, ideally using a flow diagram.	6-7, Figure 1
	16b	Cite studies that might appear to meet the inclusion criteria, but which were excluded, and explain why they were excluded.	Not available
Study characteristics	17	Cite each included study and present its characteristics.	Table 1

Section and Topic	Item #	Checklist item	Reported on page #
Risk of bias in studies	18	Present assessments of risk of bias for each included study.	37, Table S4
Results of individual studies	19	For all outcomes, present, for each study: (a) summary statistics for each group (where appropriate) and (b) an effect estimate and its precision (e.g. confidence/credible interval), ideally using structured tables or plots.	Figures 2-5, Table 2
Results of syntheses	20a	For each synthesis, briefly summarise the characteristics and risk of bias among contributing studies.	8, Figures 2-5
	20b	Present results of all statistical syntheses conducted. If meta-analysis was done, present for each the summary estimate and its precision (e.g. confidence/credible interval) and measures of statistical heterogeneity. If comparing groups, describe the direction of the effect.	24-37, Figures 2-5
	20c	Present results of all investigations of possible causes of heterogeneity among study results.	36-37
	20d	Present results of all sensitivity analyses conducted to assess the robustness of the synthesized results.	36-37, Figures S1-S4
Reporting biases	21	Present assessments of risk of bias due to missing results (arising from reporting biases) for each synthesis assessed.	Not available
Certainty of evidence	22	Present assessments of certainty (or confidence) in the body of evidence for each outcome assessed.	Not available
**DISCUSSION**			
Discussion	23a	Provide a general interpretation of the results in the context of other evidence.	37-40
	23b	Discuss any limitations of the evidence included in the review.	42
	23c	Discuss any limitations of the review processes used.	41
	23d	Discuss implications of the results for practice, policy, and future research.	42
**OTHER INFORMATION**			
Registration and protocol	24a	Provide registration information for the review, including register name and registration number, or state that the review was not registered.	3
	24b	Indicate where the review protocol can be accessed, or state that a protocol was not prepared.	3
	24c	Describe and explain any amendments to information provided at registration or in the protocol.	Not available
Support	25	Describe sources of financial or non-financial support for the review, and the role of the funders or sponsors in the review.	43
Competing interests	26	Declare any competing interests of review authors.	44
Availability of data, code and other materials	27	Report which of the following are publicly available and where they can be found: template data collection forms; data extracted from included studies; data used for all analyses; analytic code; any other materials used in the review.	43

From: Page MJ, McKenzie JE, Bossuyt PM, Boutron I, Hoffmann TC, Mulrow CD, et al. The PRISMA 2020 statement: an updated guideline for reporting systematic reviews. BMJ 2021; 372 :n71. doi: 10.1136/bmj.n71

Table S2. Search strategy through electronic databases.

**PubMed**	"Stenotrophomonas maltophilia" [Text Word] AND ("TMP-SMX"[Text Word] OR "trimethoprim-sulfamethoxazole"[Text Word] OR "cotrimoxazole"[Text Word] OR "trimethoprim"[Text Word] OR "minocycline"[Text Word] OR "tigecycline"[Text Word] OR " tetracycline*"[Text Word] OR "fluoroquinolone*"[Text Word] OR "quinolone*"[Text Word] OR "levofloxacin"[Text Word] OR "ciprofloxacin" [Text Word] OR "cefiderocol" [Text Word] OR "ceftazidime" [Text Word] OR "avibactam" [Text Word] OR "ceftazidime-avibactam" [Text Word] OR "monotherapy" [Text Word] OR "combination" [Text Word])
**EMBASE**	'stenotrophomonas maltophilia':ti,ab,kw AND ('tmp-smx':ti,ab,kw OR 'trimethoprim-sulfamethoxazole':ti,ab,kw OR 'cotrimoxazole':ti,ab,kw OR 'trimethoprim':ti,ab,kw OR 'minocycline':ti,ab,kw OR 'tigecycline':ti,ab,kw OR 'tetracycline*':ti,ab,kw OR 'fluoroquinolone*':ti,ab,kw OR 'quinolone*':ti,ab,kw OR 'levofloxacin':ti,ab,kw OR 'ciprofloxacin':ti,ab,kw OR 'cefiderocol':ti,ab,kw OR 'ceftazidime':ti,ab,kw OR 'avibactam':ti,ab,kw OR 'ceftazidime-avibactam':ti,ab,kw OR 'monotherapy':ti,ab,kw OR 'combination':ti,ab,kw) AND [01-01-1900]/sd NOT [16-03-2022]/sd

Table S3. Sensitivity analyses specifically addressing the role of the study by Sarzinsky and colleagues[44] in the comparison between TMP/SMX and FQs (primary outcome).

Outcome: Mortality (all-cause)							
**Comparison**	**Included studies**	**Number of patients**	**OR, 95% CI**	**I^2**	**Prediction interval**	**E-value**	**Comments**
TMP/SMX vs FQs	7	1953	1.48 (1.14-1.92)	43%	1.05-2.08	For point estimate: 1.73; for CI: 1.34.	Compared with the main analysis, there was the exclusion of four studies run in the United States[25,33,35,39] before the one by Sarzinsky and collaborators.[44]<br>All monotherapy studies.<br>One pediatric study.[27]<br>FQs: four studies about levofloxacin,[24,26,31,44] one about ciprofloxacin,[37] two mixed.[28,32]
TMP/SMX vs FQs - BSI	5	397	2.15 0.83-5.58	62%	0.01-50.96	For point estimate: 2.29; for CI: 1.	Compared with the main analysis, there was the addition of derived data from Sarzinsky and co-workers:[44] 13 events in the FQs arm (out of 99 patients), 10 events in the TMP/SMX arm (out of 64 subjects).<br>Different timing of mortality: 30-day,[24] in-hospital,[28,33,44] 7-day.[37]<br>One pediatric study.[37]<br>FQs: two studies about levofloxacin,[24,44] one about ciprofloxacin,[37] two mixed.[28,33]
TMP/SMX vs FQs not only monotherapy	11	2352	1.64 (1.07-2.53)	50%	0.49-5.52	For point estimate: 1.88; for CI: 1.22.	Compared with the main analysis, there was the exclusion of four studies run in the United States[25,33,35,39] before the one by Sarzinsky and collaborators.[44]<br>One pediatric study.[37]<br>FQs: five studies about levofloxacin,[24,26,31,36,44] two about ciprofloxacin,[37,43], four mixed.[28,32,34,40]
TMP/SMX vs FQs not only monotherapy - BSI	8	632	2.19 (1.12-2.48)	59%	0.30-15.84	For point estimate: 2.32; for CI: 1.31.	Compared with the main analysis, there was the addition of derived data from Sarzinsky and co-workers:[44] 13 events in the FQs arm (out of 99 patients), 10 events in the TMP/SMX arm (out of 64 subjects).<br>Different timing of mortality:<br>30-day,[24,41] in-hospital,[28,33,44] 60-day,[34] 90-day,[43] 7-day.[37]<br>One pediatric study.[37]

							FQs: three studies about levofloxacin,[24,41,44] two about ciprofloxacin,[37,43] three mixed.[28,33,34]
**Outcome: Mortality – adjusted effect size**							
**Comparison**	**Included studies**	**Number of patients**	**OR, 95% CI**	**I²**	**Prediction interval**	**E-value**	**Comments**
FQs vs TMP/SMX	2	1667	0.75 (0.57-0.99)	0%	Not calculable	For point estimate: 1.58; for CI: 1.08.	Compared with the main analysis, there was the exclusion of one study run in the United States[39] before the one by Sarzinsky and collaborators.[44] <br>All monotherapy studies.

FQs: fluoroquinolones; OR: odds ratio; TMP/SMX: trimethoprim/sulfamethoxazole

Table S4 (a). Quality assessment of studies through a modified version of the Newcastle-Ottawa Assessment Scale.

Cohort studies									
**Study**	SELECTION MAX 4				COMPARABILITY MAX 2	OUTCOME MAX 3			TOTAL STAR RATING UP TO 9
	Representativeness of the exposed cohort	Selection of the non-exposed cohort	Ascertainment of exposure	Outcome not present at start of study	Comparability of cohorts at baseline	Assessment of outcome	Follow-up long enough	Follow-up complete	Assessment of bias risk
**Garcia Paez et al., 2018**	★	★	★	★			★	★	**6 High risk of bias[a]**
**Czosnowski et al., 2011**	★	★	★	★			★	★	**6 High risk of bias[a]**
**Tekçe et al., 2012**	★	★	★	★	★		★	★	**7 Moderate risk of bias**
**Cho et al., 2014**	★	★	★	★	★		★	★	**7 Moderate risk of bias**
**Wang Y.L. et al. 2014**	★	★	★	★	★		★	★	**7 Moderate risk of bias**
**Gozel et al., 2015**	★	★	★	★			★	★	**6 High risk of bias[a]**
**Hand et al., 2016**	★	★	★	★	★		★	★	**7 Moderate risk of bias**
**Wang C.H. et al., 2016**	★	★	★	★			★	★	**6 High risk of bias[a]**
**Chen et al., 2107**	★	★	★	★			★	★	**6 High risk of bias[a]**
**Ebara et al., 2017**	★	★	★	★			★	★	**6 High risk of bias[a]**
**Kim S.H. et al.**, 2018	★	★	★	★	★		★	★	**7 High risk of bias[a]**
**Velázquez-Acosta et al., 2018**	★	★	★	★	★		★	★	**7 High risk of bias[a]**
**Watson et al., 2018**	★	★	★	★	★		★	★	**7 Moderate risk of bias**
**Kim E.J. et al., 2018**	★	★	★	★			★	★	**6 High risk of bias[a]**
**Nys et al., 2019**	★	★	★	★	★		★	★	**7 Moderate risk of bias**
**Shah et al., 2019**	★	★	★	★	★		★	★	**7 Moderate risk of bias**
**Tokatly Latzer et al., 2019**	★	★	★	★	★		★	★	**7 High risk of bias[a]**
**Alsuhaiban et al., 2021**	★	★	★	★			★	★	**6 High risk of bias[a]**
**Junco et al., 2021**	★	★	★	★	★		★	★	**7 Moderate risk of bias**
**Puech et al., 2021**	★	★	★	★	★		★	★	**7 High risk of bias[a]**
**Tuncel et al., 2021**	★	★	★	★	★		★	★	**7 High risk of bias[a]**
**Zha et al., 2021**	★	★	★	★	★		★	★	**7 Moderate risk of bias**
**Ahlstrom et al, 2022**	★	★	★	★	★		★	★	**7 High risk of bias[a]**
**Sarzynski et al., 2022**	★	★	★	★	★ ★			★	**8 Low risk of bias**

★

Case-control studies									
**Study**	SELECTION MAX 4				COMPARABILITY MAX 2	OUTCOME MAX 3			TOTAL STAR RATING UP TO 9
	Adequacy of case definition	Cases representative of the population under investigation	Selection of the controls	Selection of the controls	Comparability of cases and controls at baseline	Assessment of exposure	Same method to ascertain cases and controls	Missing rate	Assessment of bias risk
\	\	\	\	\	\	\	\	\	\

Notes:
[a] Downgrade due to study design, originally conceived not compare different antibiotic regimens for *Stenotrophomonas maltophilia* infection

Table S4 (b). Definition for the adapted version of the Newcastle-Ottawa Assessment Scale used to the purposes of the present review.

Cohort studies		Criteria to fulfill
Category: Selection	Representativeness of the exposed cohort	Cases of *Stenotrophomonas maltophilia* infection exposed to a reference drug/regimen
	Selection of the non exposed cohort	Cases of *Stenotrophomonas maltophilia* infection exposed to an alternative drug/regimen drawn from the same population of the exposed to the reference drug/regimen
	Assessment of exposure	Secure records
	Demonstration that the outcomes of interest were not present at the study start	Yes
Category: Comparability	Comparability of cohorts on the basis of design or analysis	If propensity score matching: two stars
		Other matching methods involving at least criteria among age, severity of illness, comorbidity burden (e.g. Charlson Score Index): one star; alternatively, overlapping baseline features between groups
Category: Outcome	Assessment of outcome	Prospective collection of data or record linkage
	Long enough follow-up	At least 30 day
	Adequacy of follow-up	Complete data for all subjects accounted for or limited loss to follow-up (maximum 10%)
Case-control studies		
Category: Selection	Adequacy of case definition	*Stenotrophomonas maltophilia* infection exposed to a reference drug/regimen
	Representativeness of the cases	Consecutive or obviously representative series of cases
	Selection of the controls	Controls from the same population of cases
	Definition of controls	Subjects with *Stenotrophomonas maltophilia* infection receiving an alternative to the reference drug/regimen
Category: Comparability	Comparability of cases and controls on the basis of design or analysis	Study controls for age and severity of illness: one star
		Study controls for any additional factor (e.g., comorbidity burden): another star
Category: Exposure	Ascertainment of exposure	Secure records
	Same method of ascertainment for cases and controls	Yes

	Missing rate	Same for both groups

Notes: A study can be awarded a maximum of one star for each item as for the Selection and Outcome categories; a maximum of two stars con be given for Comparability.
Abbreviations: BSI: bloodstream infection.

Figure S1. Meta-analysis regarding the contrast between TMP/SMX and FQs in the setting of monotherapy, stratifying according to the comparative nature of the studies (primary outcome).

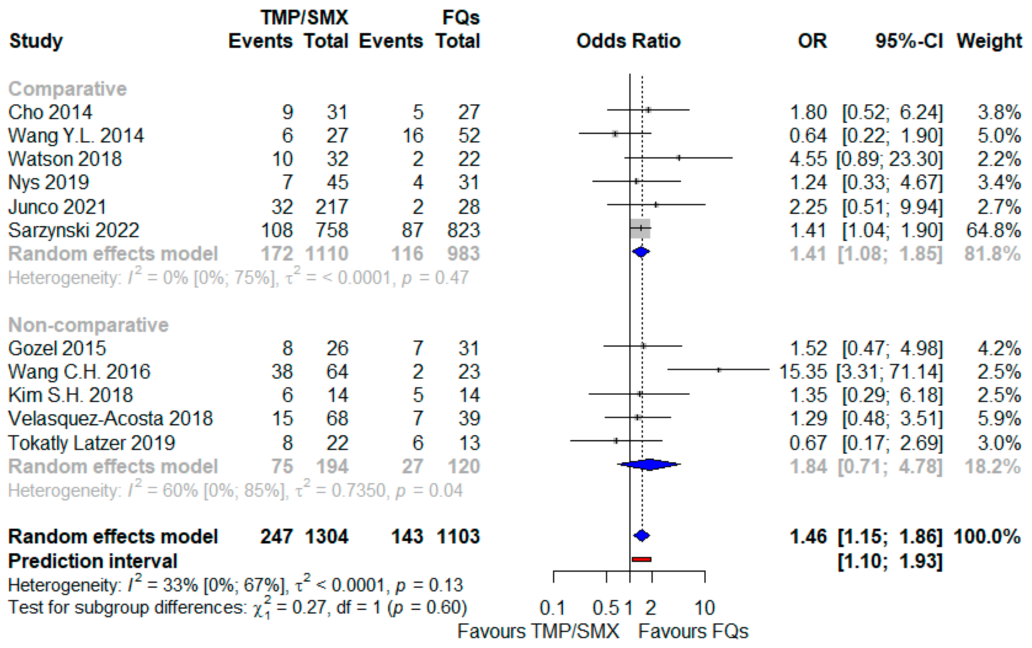

Abbreviations: FQs: fluoroquinolones; OR: odds ratio; TMP/SMX: trimethoprim/sulfamethoxazole.

Figure S2. Meta-analysis regarding the contrast between TMP/SMX and FQs in the setting of not only monotherapy, stratifying according to the comparative nature of the studies (primary outcome).

Study	TMP/SMX Events	TMP/SMX Total	FQs Events	FQs Total	OR	95%-CI	Weight
**Comparative**							
Cho 2014	14	51	7	35	1.51	[0.54; 4.25]	7.3%
Wang Y.L. 2014	6	27	16	52	0.64	[0.22; 1.90]	6.9%
Watson 2018	10	32	2	22	4.55	[0.89; 23.30]	3.8%
Nys 2019	7	45	4	31	1.24	[0.33; 4.67]	5.3%
Junco 2021	32	217	2	28	2.25	[0.51; 9.94]	4.4%
Sarzynski 2022	108	758	87	823	1.41	[1.04; 1.90]	16.2%
Random effects model	177	1130	118	991	1.40	[1.08; 1.83]	44.0%
Heterogeneity: I^2 = 0% [0%; 75%], τ^2 = < 0.0001, p = 0.49							
**Non-comparative**							
Gozel 2015	8	26	7	31	1.52	[0.47; 4.98]	6.1%
Wang C.H. 2016	38	64	2	23	15.35	[3.31; 71.14]	4.2%
Kim S.H. 2018	6	14	5	14	1.35	[0.29; 6.18]	4.3%
Velasquez-Acosta 2018	15	68	7	39	1.29	[0.48; 3.51]	7.6%
Kim E.J. 2019	27	31	19	40	7.46	[2.20; 25.26]	5.9%
Tokatly Latzer 2019	8	22	6	13	0.67	[0.17; 2.69]	4.9%
Puech 2021	40	80	44	84	0.91	[0.49; 1.68]	11.9%
Tunchel 2021	18	49	4	17	1.89	[0.53; 6.67]	5.6%
Ahlstrom 2022	9	48	4	22	1.04	[0.28; 3.82]	5.4%
Random effects model	169	402	98	283	1.80	[0.97; 3.34]	56.0%
Heterogeneity: I^2 = 60% [16%; 81%], τ^2 = 0.5163, p = 0.01							
**Random effects model**	**346**	**1532**	**216**	**1274**	**1.58**	**[1.10; 2.27]**	**100.0%**
**Prediction interval**						**[0.58; 4.35]**	

Heterogeneity: I^2 = 43% [0%; 69%], τ^2 = 0.1853, p = 0.04

Test for subgroup differences: χ^2_1 = 0.52, df = 1 (p = 0.47)

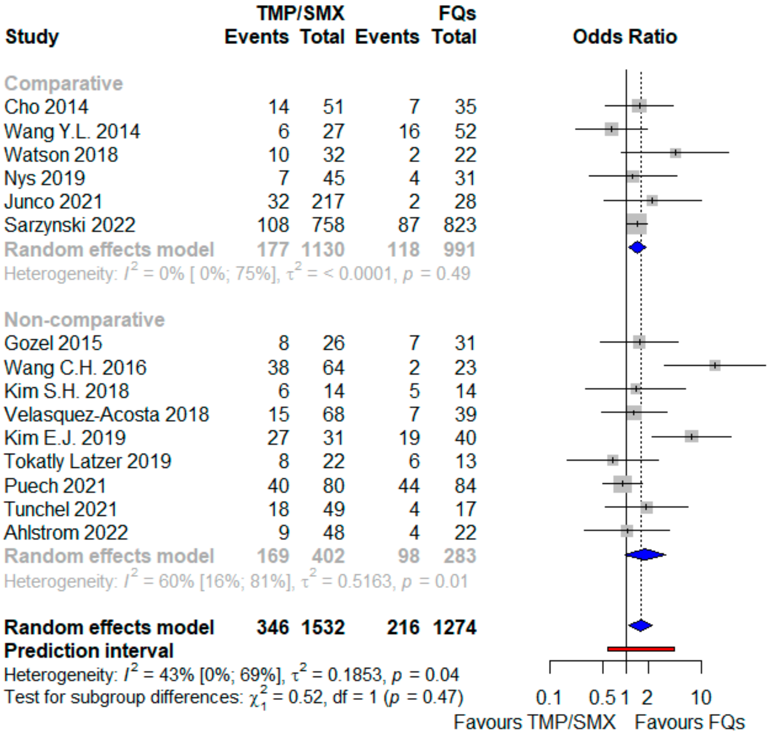

Abbreviations: FQs: fluoroquinolones; OR: odds ratio; TMP/SMX: trimethoprim/sulfamethoxazole.

Figure S3. Influential plot visualizing the summary effect sizes and heterogeneity values (I^2) for meta-analyses without the study named in each row regarding the comparison between TMP/SMX and FQs in the setting of monotherapy (primary outcome).

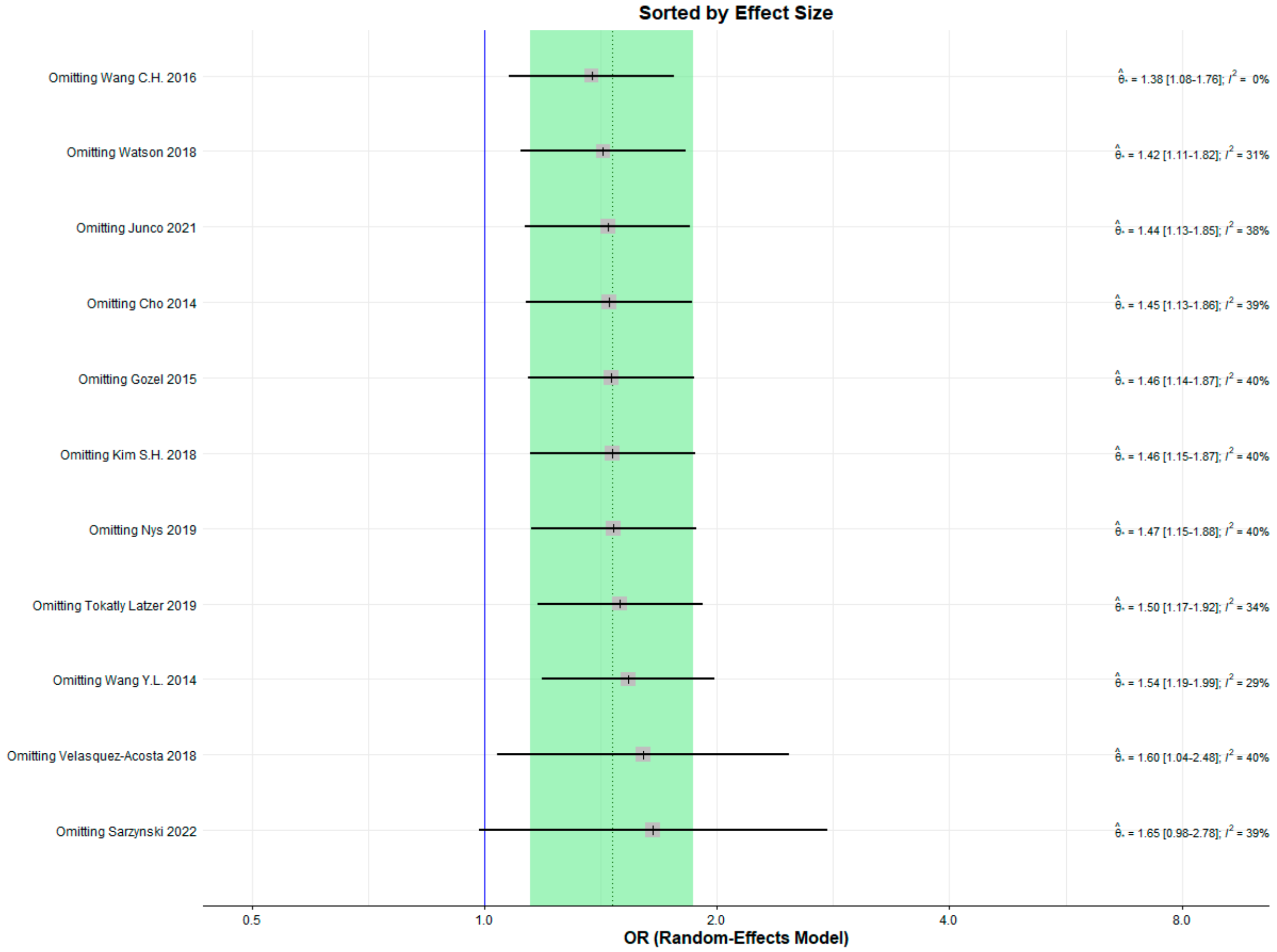

Abbreviations: FQs: fluoroquinolones; TMP/SMX: trimethoprim/sulfamethoxazole.

Figure S4. Influential plot visualizing the summary effect sizes and heterogeneity values (I^2) for meta-analyses without the study named in each row regarding the comparison between TMP/SMX and FQs in the setting of not only monotherapy (primary outcome).

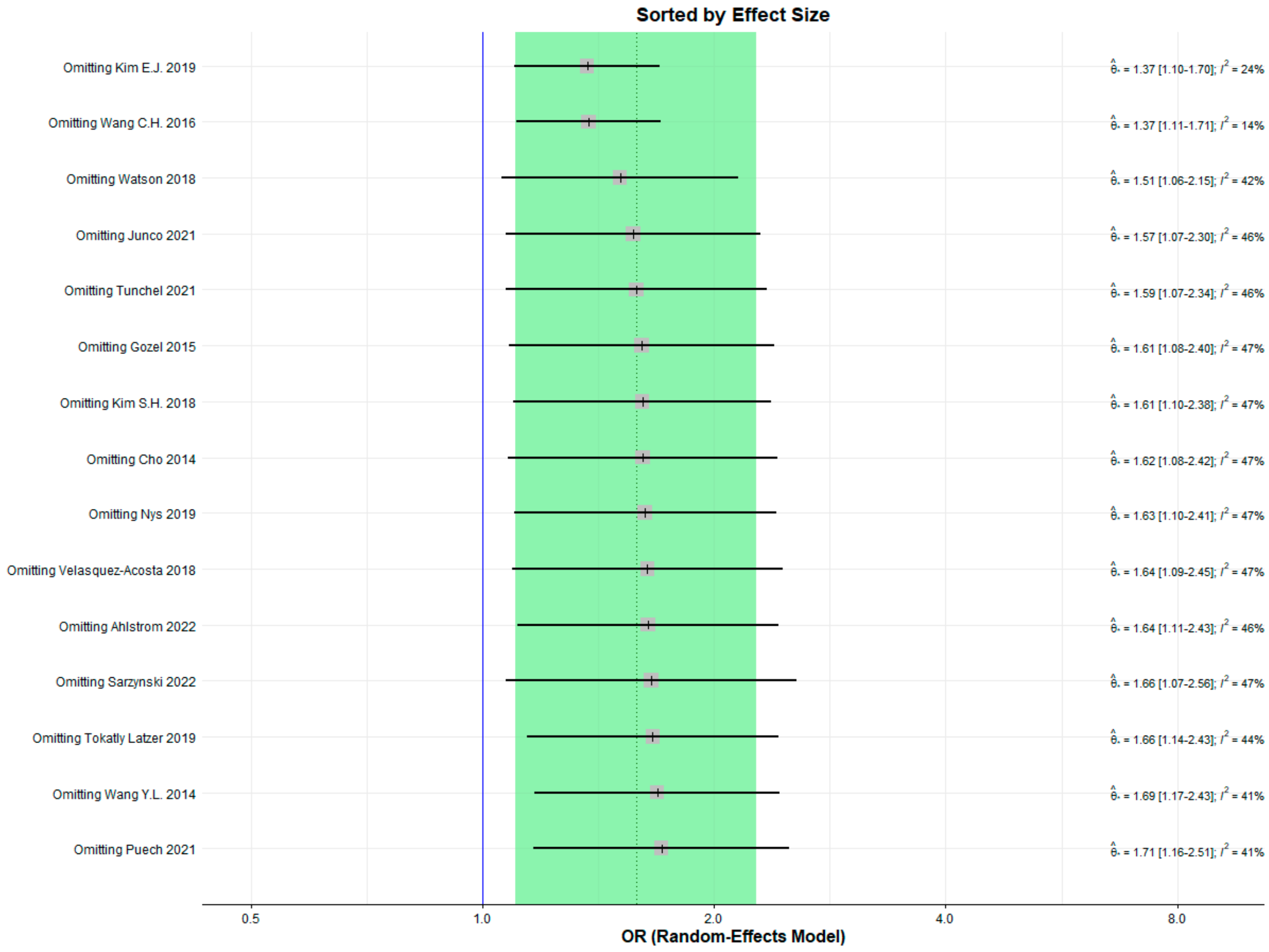

Abbreviations: FQs: fluoroquinolones; TMP/SMX: trimethoprim/sulfamethoxazole.

Figure S5. Contour-enhanced funnel plot concerning the comparison between TMP/SMX and FQs in the setting of only monotherapy (primary outcome).

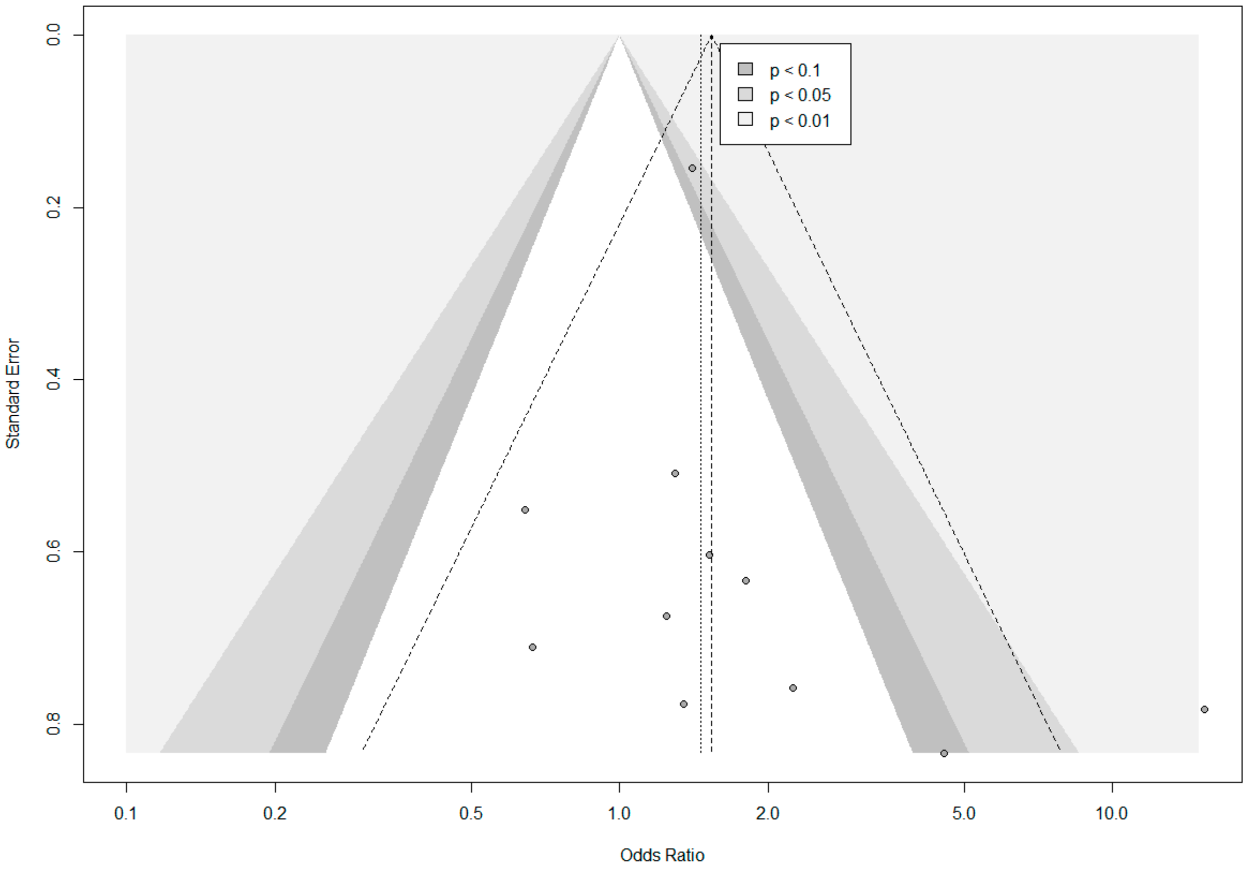

Abbreviations: FQs: fluoroquinolones; TMP/SMX: trimethoprim/sulfamethoxazole.

Figure S6. Contour-enhanced funnel plot concerning the comparison between TMP/SMX and FQs in the setting of not only monotherapy (primary outcome).

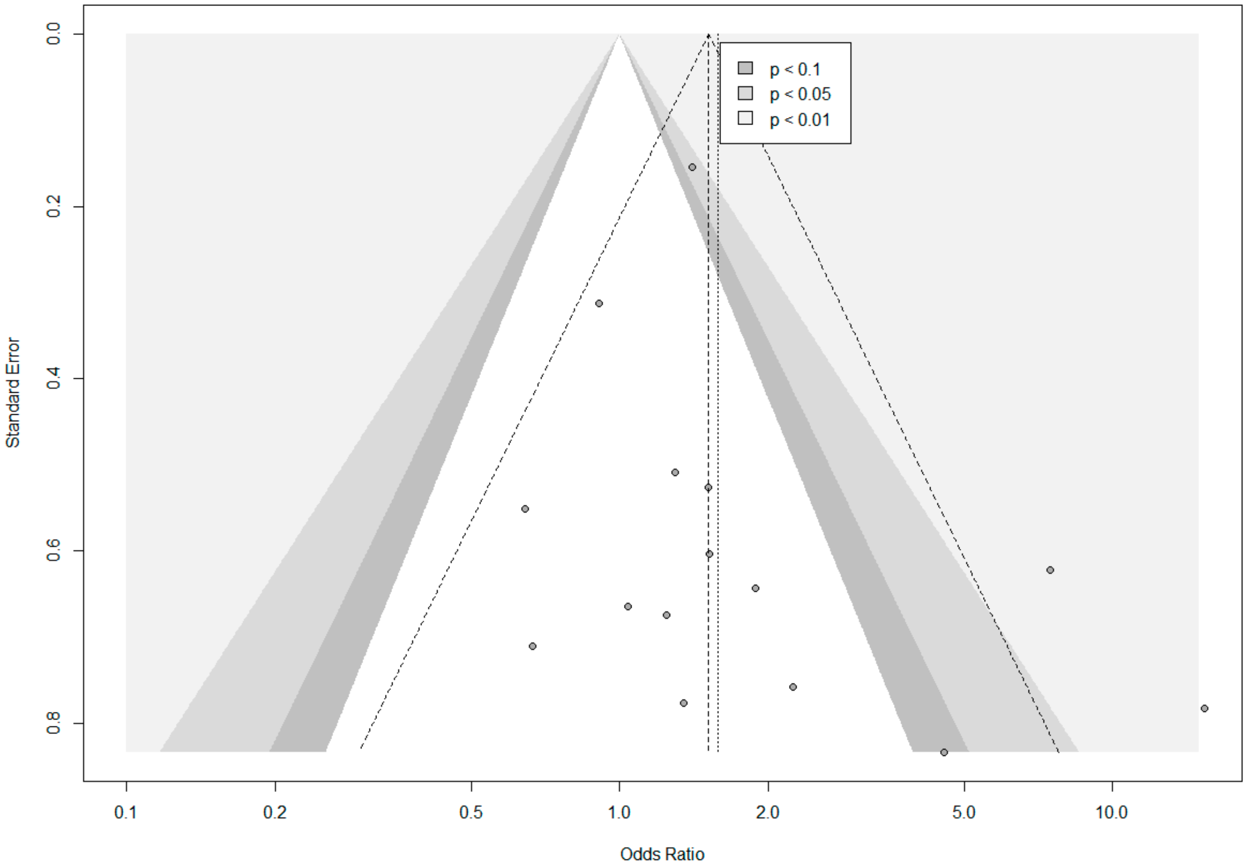

Abbreviations: FQs: fluoroquinolones; TMP/SMX: trimethoprim/sulfamethoxazole.
